# Editorial: Vascular Disease Multi-Scale Multi-Physics Modeling and Experimental Data

**DOI:** 10.3389/fphys.2022.865905

**Published:** 2022-03-17

**Authors:** Sanjay R. Kharche, Halina Dobrzynski, Daniel Goldman

**Affiliations:** ^1^Department of Medical Biophysics, Western University, London, ON, Canada; ^2^Division of Cardiovascular Sciences, University of Manchester, Manchester, United Kingdom; ^3^Kidney Clinical Research Unit, Lawson Health Research Institute, London, ON, Canada; ^4^Department of Anatomy, Jagiellonian University Medical College, Kraków, Poland

**Keywords:** large data, vascular disease, multi-physics modeling, multi-scale computer modeling, collaboration

## Modern Experimental Methods Necessitate Data Integration and Interpretation

Model experimental research generates large data. Technology now permits multi-scale investigations from sub-molecular to whole specimens (Evans et al., 2021). The Dobrzynski group use a combination of experimental-imaging methods to uncover crucial relationships between cardiac structure and function (Dobrzynski and Boyett, 2006; Logantha et al., 2016) to advance our understanding of whole heart pathophysiology.

In this Research Topic, Yin et al. succinctly show how integrating multi-scale experimental data scaffolded by mechanistic computational modeling has led them to a novel strategy wherein augmenting the number of smooth muscle cells in recovering skeletal muscle capillaries may treat peripheral arterial disease. The method and techniques work by Liu et al. provides a stable and minimally invasive rabbit model of cerebral stenosis that permits investigation of underlying whole body hemodynamic processes in a physiologically informative manner.

We believe that data driven multi-scale computational modeling is a robust approach to integrate experimental knowledge and gain insights into key pathophysiological processes.

## Multi-Scale Computational Modeling Integrates Experimental Knowledge

Computational modeling provides a quantitative paradigm to assess whether individual experimental findings “fit into a whole.” The Goldman group combine microcirculation and organism level measurements to predict oxygen transport in skeletal muscle [see e.g., Farid et al. (2017)]. The Kharche laboratory uses experimental-clinical data to test otherwise intractable hypothesis such as cardiac sino-atrial node exit pathways and presence of peripheral arterial disease (Kharche et al., 2017, 2018) to further clarify existing scientific evidence.

In this Research Topic, Naber et al. have deployed computational fluid dynamics (CFD) and data analysis to reduce the quantitative uncertainty in calculating vessel transit time, accurate measurement of which may improve brain surgery outcomes. The work by Ai et al. computes coronary microvascular resistance using a combination of non-invasive angiography imaging and CFD modeling leveraged by their prior findings, an approach that may alleviate the use of risk augmenting invasive wires. Hashemi et al. used CFD to compute a spectrum of hemodynamic parameters (e.g., wall shear stress, residence time) that allows stratification of the severity of atherosclerosis plaque driven stenosis, which may provide insights into smooth muscle cell and sub-cellular pathophysiological processes. The Tamis and Drapaca vascular tone model showed that an increased vessel wall stiffness is simultaneous to unavailability of important messengers such as nitrous-oxide, a description that can easily become incorporated into more detailed simulations upon suitable parameter identification. The multi-scale nephron model presented by Swapnasrita et al. strongly suggests that male and female kidneys respond differentially to diseases (diabetes) and pharmacological treatments (SGLT2 inhibition) due to the differential expression of sex specific transporters, a finding that will streamline future animal experiments and clinical trials. The machine learning work presented in this Research Topic (Bizjak et al.) shows the relevance of deep data inquiry (i.e., aneurysm sphericity, size, and volume) to enable reliable cerebral aneurysm rupture risk prediction, a modeling approach that is expected to find extensive application in the wider large data ecosystem.

## Deeper Collaboration as an Unmet Need

It can be appreciated that experimentalists, modelers, and clinicians are traditionally considered to be end users of each other's knowledge. We believe that an important factor in translation is a deeper inter-field engagement (Yoda, 2016) which may lead to methods refinement, accelerated research outcomes, as well as synergize knowledge exploitation to improve human and animal quality of life.

## Author Contributions

SK wrote the editorial, which was revised and approved by DG and HD. All authors contributed to the article and approved the submitted version.

## Funding

This work was supported by Canada's Canarie Inc (RS3-111).

## Conflict of Interest

The authors declare that the research was conducted in the absence of any commercial or financial relationships that could be construed as a potential conflict of interest.

## Publisher's Note

All claims expressed in this article are solely those of the authors and do not necessarily represent those of their affiliated organizations, or those of the publisher, the editors and the reviewers. Any product that may be evaluated in this article, or claim that may be made by its manufacturer, is not guaranteed or endorsed by the publisher.
